# A Frequency Reconfigurable MIMO Antenna with Bandstop Filter Decoupling Network for Cognitive Communication

**DOI:** 10.3390/s22186937

**Published:** 2022-09-14

**Authors:** Hashinur Islam, Saumya Das, Tanweer Ali, Tanushree Bose, Om Prakash, Pradeep Kumar

**Affiliations:** 1 Department of Electronics and Communication Engineering, Sikkim Manipal Institute of Technology, Sikkim Manipal University, Gangtok 737136, India; 2 Department of Information Technology, Sikkim Manipal Institute of Technology, Sikkim Manipal University, Gangtok 737136, India; 3 Department of Electronics and Communication Engineering, Manipal Institute of Technology, Manipal Academy of Higher Education, Manipal 576104, India; 4 ECE Department, Sri Venkateswara College of Engineering & Technology, Chittoor 517127, India; 5 Discipline of Electrical, Electronic and Computer Engineering, University of KwaZulu-Natal, Durban 4041, South Africa

**Keywords:** reconfigurable MIMO antenna, bandstop filter-based decoupling network, cognitive communication system

## Abstract

A compact reconfigurable MIMO antenna was developed for cognitive radio applications in this research work. A bandstop filter-based decoupling network was employed in this MIMO antenna to keep mutual coupling at a minimum. A single PIN diode was connected in the filter configuration for the purpose of reconfiguration. Controlling the ON/OFF conditions of the PIN diode, it became possible to achieve a MIMO operating frequency of 4.75 GHz in mode 1 and 1.77 GHz in mode 2, respectively. At 4.75 GHz, isolation was 42.68 dB, while at 1.77 GHz, isolation was 26.52 dB. The proposed reconfigurable MIMO antenna achieved a peak gain and radiation efficiency of 6.63 dBi and 92.04 percent in mode 1 and 4.41 dBi and 89.64 percent in mode 2. MIMO characteristics such as an envelope correlation coefficient (ECC) less than 0.253, diversity gain (DG) greater than 9.675 dB, a mean effective gain (MEG) measurement ratio of less than 0.00388 dB, and channel capacity loss (CCL) of less than 0.06528 bits/s/Hz were measured for both operational frequency bands. To make it simple to integrate into small wireless devices, the overall size of the antenna is limited to $48\;\mathrm{mm}\times 24\;\mathrm{mm}\;\left(0.28\;\lambda_{0}\times 0.12\;\lambda_{0}\right)$.

## 1. Introduction

Because of the rapid growth of wireless communications, the spectrum has become extremely congested. Cognitive communication has attracted a lot of attention over the last two decades as a way to increase spectrum utilization. Cognitive radio (CR) has emerged as a game-changing technique for utilizing the scarce frequency spectrum in a dynamic, efficient, and flexible manner [1]. There are two categories of spectrum users in the CR system: (1) The primary user is the one who owns the spectrum and has first priority in using it. (2) The spectrum is accessible to the secondary user when the principal user is not available. Thus, the cognitive communication system should be able to use the frequency spectrum in a flexible manner by adjusting its frequency in response to spectrum availability and system demands. To implement this kind of communication, a frequency reconfigurable antenna is required for successful transmission and reception. The antenna operating frequency used by the primary or secondary user can be changed with a frequency reconfigurable antenna. In addition to efficient spectrum utilization, cognitive communication systems also require high system throughput, improved channel capacity, and reliability. Thus, the frequency reconfigurable antenna used in cognitive communication should also meet the high data rate requirement and efficiently use communication channels. The aforementioned challenges can be addressed by exploring frequency reconfigurable MIMO (Multiple-Input Multiple-Output) antennas for cognitive radio applications. Over the last decade, the huge trend of cognitive communication services has motivated many researchers to come up with novel and inventive ways to develop frequency reconfigurable MIMO antennas. Patch antennas have been widely employed in the development of MIMO antennas due to their low-profile structure, low cost, ease of fabrication, and simple integration mechanism [2,3]. Frequency reconfigurability was achieved in these antennas mostly by the use of PIN diodes as an ON/OFF switch. Controlling the current path by ON/OFF switching and reconfiguring the operating frequency was achieved for these antennas. However, designing reconfigurable MIMO antennas into a small and compact structure is difficult due to different challenges [4]. The presence of multiple antennas within a small space causes an increase in mutual coupling [5]. High mutual coupling significantly degrades the MIMO antenna performance. In addition to mutual coupling, the presence of PIN diodes and their corresponding biasing circuitry in the reconfigurable antenna can change antenna characteristics in real scenarios. Therefore, the number of PIN diodes in the reconfigurable antenna should be kept to a minimum. All of these issues of reconfigurable MIMO antennas were carefully considered in the present work.

To reduce mutual coupling, techniques such as decoupling networks [6], parasitic elements [7], neutralization lines [8], metasurfaces [9], metamaterial structures [10], defective ground planes [11], and bandstop filters [12] have been widely used in MIMO antennas. PIN diodes have been extensively used as switching devices for developing reconfigurable antennas [13]. PIN diodes are indeed a cheaper, faster, and more compact alternative to other switches. In reconfigurable antenna designs, PIN diodes are preferred because they allow quick dynamic reconfiguration and have switching speeds ranging from 1 to 100 nsec [14]. However, if a MIMO antenna is combined with a PIN diode, the total size of the antenna will increase, and the antenna performance may be altered as the number of PIN diodes increases [15]. Therefore, it is necessary to take into consideration how to make a portable reconfigurable MIMO antenna in a tiny wireless device with a restricted number of PIN diodes.

Some relevant research articles on reconfigurable MIMO antennas were reviewed to explore the existing work. By performing experiments with various techniques, these antennas were shown to precisely handle both reconfiguration and isolation. PIN diodes were utilized in [16] for a two-port MIMO antenna to implement frequency reconfiguration with overall dimensions of 80 mm×40 mm. Two PIN diodes are integrated with the antennas to alter the operational frequency. The resonance frequency fluctuates across WLAN bands (2400–2483 and 5150–5350 MHz) and mWiMAX bands (3400–3600 MHz) based on the ON or OFF condition of the PIN diode. On the ground plane, an isolation improvement structure (IIS) was developed to minimize the coupling current between two antennas and provide maximum isolation of 25 dB throughout all operational frequency bands.

PIN diode-based reconfigurable techniques were also used in [17] to develop UWB/WiMAX MIMO antennas with frequency bands of 3–12 GHz and 2.32–3.8 GHz. To switch antenna functioning, two PIN diodes are integrated with the antenna. When the PIN diode is turned on, the antenna produces a notch for the WiMAX 3.5 GHz (3.2–3.8 GHz) frequency band; however, when it is off, the antenna delivers the entire UWB band without band rejection characteristics. On the ground plane, a defected rectangular stub line structure was designed to improve decoupling between antenna elements, providing isolation of more than 25 dB over the entire UWB band. The overall dimensions of the two-port MIMO antenna are 14 mm × 30 mm.

The development of a compact frequency reconfigurable MIMO antenna employing four PIN diodes for microwave sensing applications in WLAN and WiMAX frequency bands is described in [18]. The designed antenna can be switched between three single frequency bands, including WLAN bands 2.42–2.5 and 5.28–5.98 GHz, as well as WiMAX band 3.41–3.59 GHz. Isolation of more than 25 dB between the antenna elements was accomplished in all three frequency bands using an inverted-T-shaped slot and subsequently a U-shaped metallic strip on the ground plane. The designed MIMO antenna has a total size of 60 mm × 20 mm, with an 8 mm edge-to-edge gap between the elements.

The authors of [19] presented a multiband frequency reconfigurable WLAN MIMO antenna that can operate in triple and dual bands. The essential switching is accomplished by two PIN diodes mounted on the two antenna elements. The developed antenna operates at 2.4, 5.2, and 5.8 GHz resonant frequencies when PIN diodes D1 and D2 are turned on and at 5.2 and 5.8 GHz resonant frequencies when PIN diodes D1 and D2 are turned off. To reduce mutual coupling, a meander line resonator and T slot decoupling network are utilized, and good isolation of 20 dB is accomplished at all operating frequencies with the help of decoupling circuits. The MIMO antenna was built on a 1.6 mm thick FR4 Epoxy substrate with dimensions of 77.5 mm × 52 mm.

In [20], PIN diode-based reconfigurable techniques were employed to create a MIMO antenna with frequency bands 2.43–2.60 GHz and 3.51–3.79 GHz. A reconfigurable bandstop filter acts as a decoupling network between two closely separated monopole antenna elements, eliminating mutual coupling and allowing the antenna parameters to be reconfigured. Two PIN diodes are included in the antenna for switching antenna functionality. When PIN diodes D1 and D2 are turned on, the developed antenna operates at the 2.43–2.60 GHz frequency band, and when PIN diodes D1 and D2 are turned off, it operates at the 3.51–3.79 GHz frequency band. In both modes, maximum isolation of more than 30 dB was obtained with the help of a decoupling network between the antenna parts. The total size of the antenna is 44 mm × 22 mm.

Therefore, the authors have the genuine goal of enhancing isolation, decreasing the number of PIN diodes, and reducing the overall size of reconfigurable MIMO antennas. In this article, we describe switchable bandstop filter-based mutual coupling reduction for a miniaturized electronically reconfigurable MIMO antenna. To eliminate the coupling current between two antennas and improve isolation, a reconfigurable bandstop filter was developed for a decoupling network between two antennas. The overall antenna structure occupies a volume of 48 mm × 24 mm and was printed on FR-4-printed circuit boards. To make a switchable bandstop filter, only one PIN diode was used, and the MIMO antenna can resonate at 4.75 GHz and 1.77 GHz, respectively, in mode 1 and mode 2 operations, with isolation of 42.68 dB and 26.52 dB, depending on the ON/OFF condition of the PIN diode. It also has adequate diversity metrics, including the envelope correlation coefficient (ECC), diversity gain (DG), mean effective gain (MEG), and channel capacity loss (CCL). The results of the simulation were compared to those of measurements. The proposed reconfigurable MIMO antenna was compared with other relevant work to determine significance. It was found that the antenna exhibits exceptionally high isolation in one of its operating frequencies, and it uses a single PIN diode to achieve two operating frequencies for the MIMO antenna.

## 2. Antenna Configuration and Design Process

To construct the reconfigurable MIMO antenna, four steps were considered:

A. Single antenna design;
B. MIMO antenna design;
C. Reconfigurable bandstop filter design as a decoupling structure;
D. MIMO antenna with reconfigurable bandstop filter design.

These steps are discussed in detail in the following section.

### 2.1. Single Antenna Design

**Figure 1:**
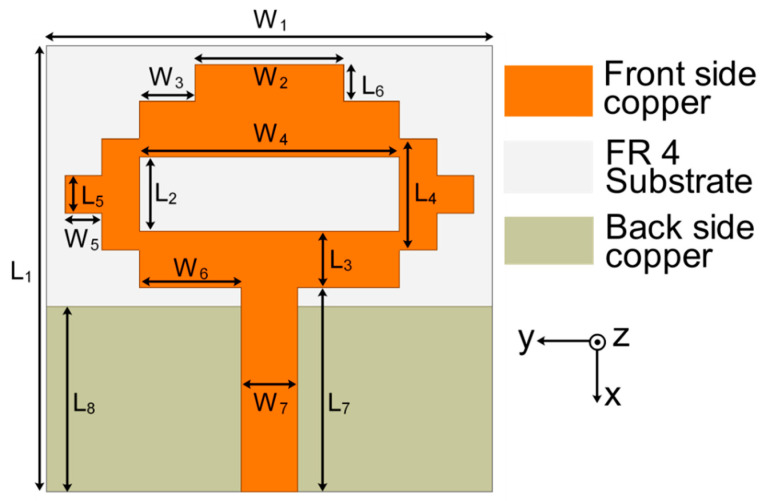
Configuration of single antenna.

**Figure 2:**
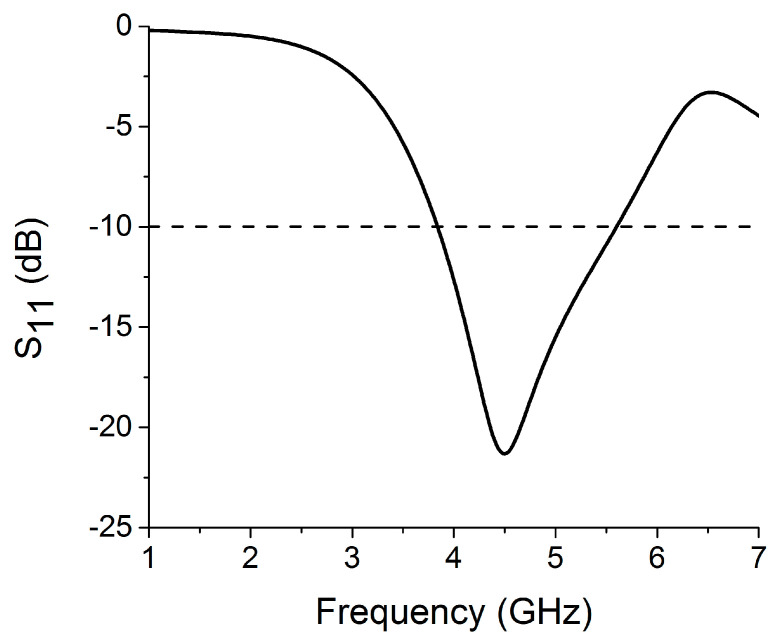
Single antenna’s simulated reflection coefficient.

**Figure 3:**
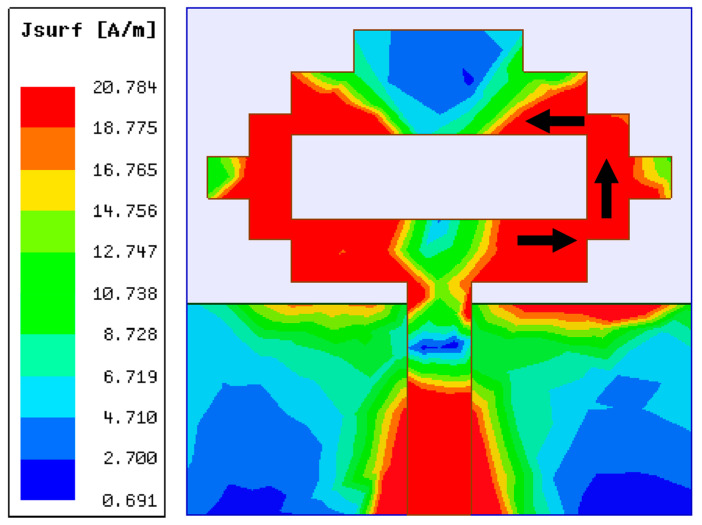
Single antenna element’s current distribution at 4.75 GHz.

In the first step of the design process, a single antenna was configured, which can be seen in Figure 1. The proposed antenna design was simulated on a 1.6 mm thick FR4 substrate with $\varepsilon_{r}=4.4$ and loss tangent tanδ = 0.02. A pixel-shaped antenna structure with a partial ground plane was designed to achieve resonance at the necessary frequency bands. The concept of a pixel-like structure for the antenna design was taken from [21,22]. The single antenna structure resonates at 4.75 GHz with a frequency range of 3.84–5.60 GHz, as shown by the reflection coefficient (Figure 2). The antenna was built on a substrate with dimensions of 24 mm × 24 mm. The design parameters of the proposed antenna are W_1_ = 24, W_2_ = 8, W_3_ = 3, W_4_ = 14, W_5_ = 2, W_6_ = 5.5, W_7_ = 3, L_1_ = 24, L_2_ = 4, L_3_ = 3, L_4_ = 6, L_5_ = 2, L_6_ = 2, L_7_ = 11, and L_8_ = 10 (unit: mm). With this monopole antenna configuration, the microstrip feed line extends from the center to achieve the appropriate current path for supporting a center frequency of 4.75 GHz. Using the quarter-wavelength (*λ*/4) monopole antenna concept, the current distribution of the antenna in Figure 3 reveals a current path length of 16 mm, which leads the structure to resonate at 4.75 GHz. The partial ground plane of the monopole antenna contributes to achieving better impedance matching. To optimize the sizes of single antenna design parameters, a rigorous simulation was performed on the HFSS platform.

### 2.2. MIMO Antenna Design

**Figure 4:**
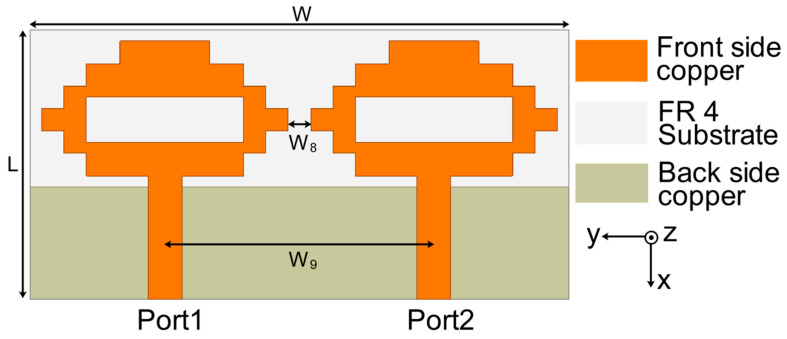
MIMO antenna construction of two elements.

**Figure 5:**
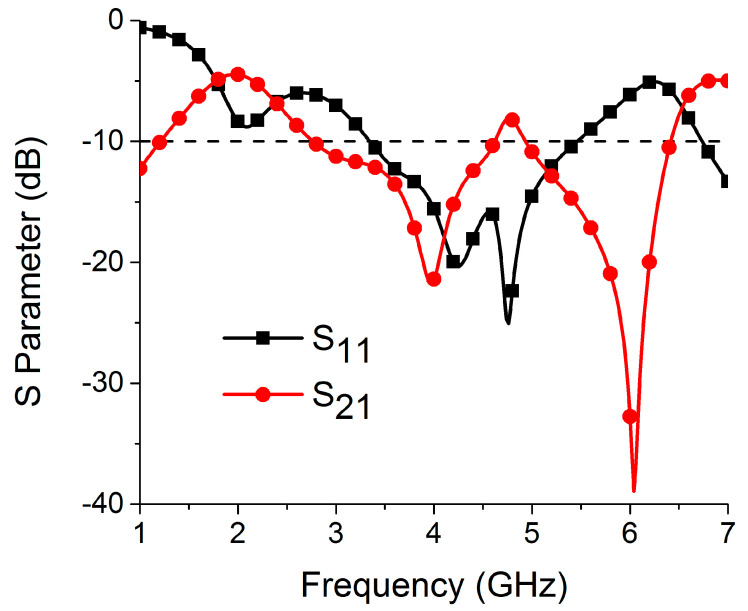
MIMO antenna S-parameters with two elements.

**Figure 6:**
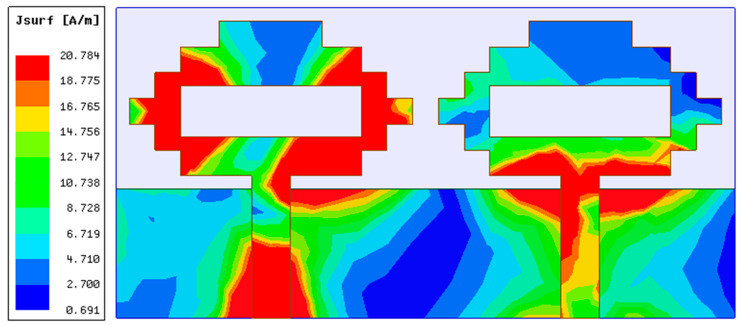
Two-element MIMO antenna’s current distribution for 4.75 GHz.

In the second part of the design process, a two-port MIMO antenna was constructed, as illustrated in Figure 4, by combining two single antenna structures together in a small space with dimensions of 48 mm × 24 mm. The resonance at 4.75 GHz (3.35–5.46 GHz) is confirmed by the simulation results of S-parameters shown in Figure 5. This figure also demonstrates strong mutual coupling between the two ports. Because of the strong mutual coupling between both antenna components and the ground plane, the antenna is unable to deliver the best performance. When port 1 is turned on and port 2 is connected to a 50 ohm matched load, the surface current distribution displayed in Figure 6 further highlights the significant mutual coupling with other antenna elements as well as the ground plane. The edge-to-edge gap (W_8_) between two antenna elements is preserved at 2 mm, while the center-to-center gap (W_9_) is kept at 24 mm.

### 2.3. Reconfigurable Bandstop Filter Design as Decoupling Structure

**Figure 7:**
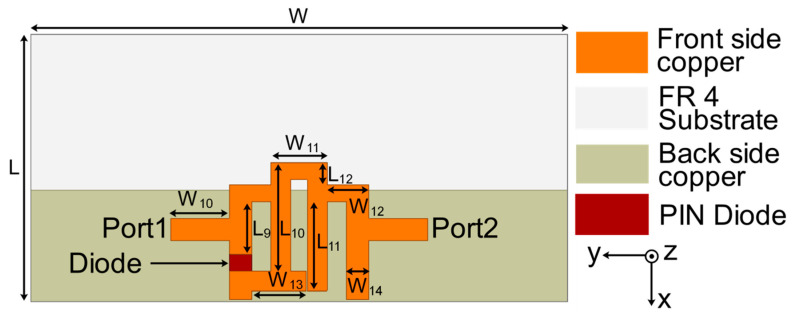
Bandstop filter construction with a single PIN diode.

**Figure 8:**
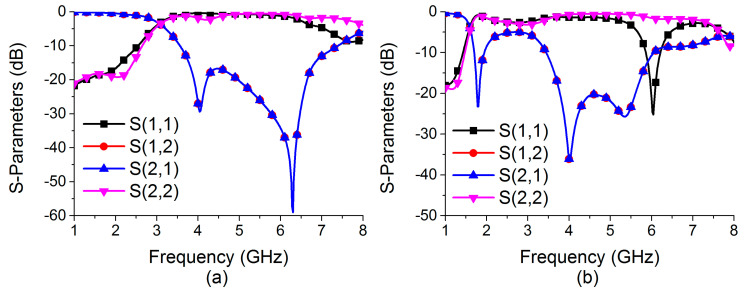
Characteristics of the bandstop filter in (**a**) mode 1 and (**b**) mode 2.

**Figure 9:**
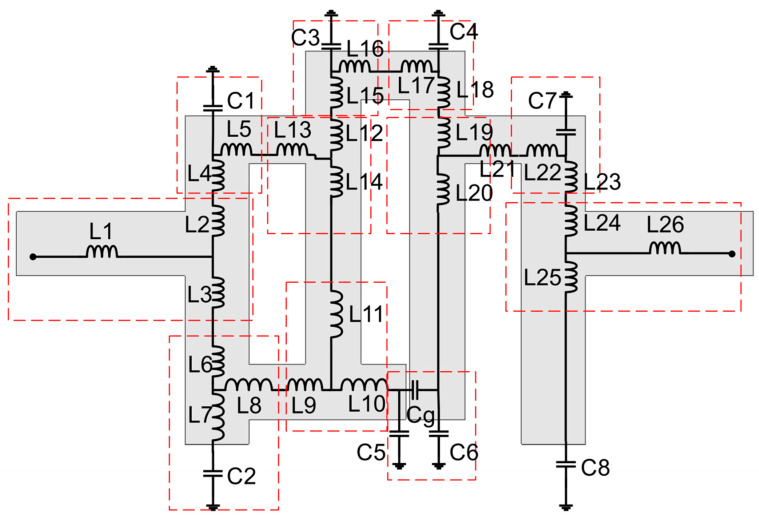
Equivalent circuit when PIN diode is ON (mode 1).

**Figure 10:**
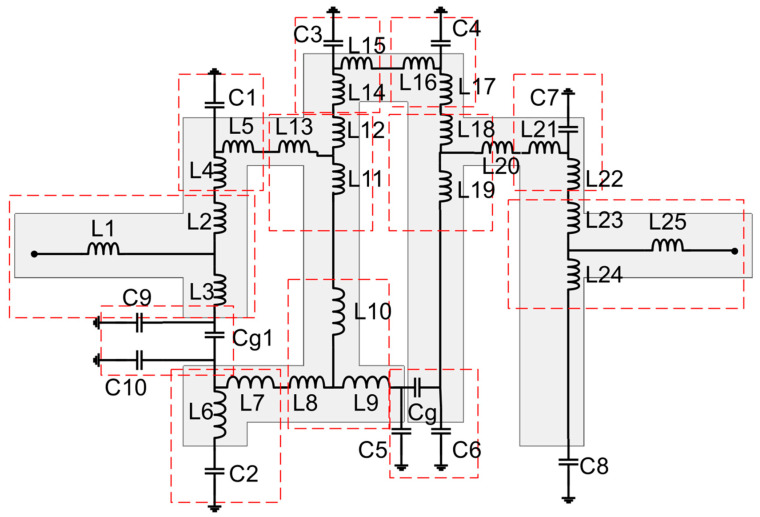
Equivalent circuit when PIN diode OFF (mode 2).

**Figure 11:**
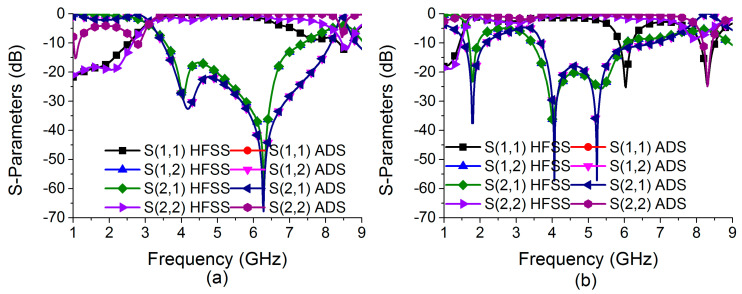
Comparison of HFSS and equivalent circuit model simulations in (**a**) mode 1 and (**b**) mode 2.

Efforts are being made to construct a reconfigurable decoupling network to reduce coupling in MIMO antennas during this phase of development. Because of this, a two-state switchable bandstop filter based on a single PIN diode was designed on the FR4 substrate depicted in Figure 7. The following are the design parameters for the reconfigurable bandstop filter: W = 48, W_10_ = 4.25, W_11_ = 5, W_12_ = 3.75, W_13_ = 4.9, W_14_ = 2, L = 24, L_9_ = 4.75, L_10_ = 9.75, L_11_ = 8, and L_12_ = 2 (unit: mm). All parameter values are optimized to achieve the required characteristics at the desired frequencies. Even a small change in these parameter values significantly deteriorates the bandstop filter operation. When the PIN diode is turned ON, the switchable bandstop filter can perform a bandstop operation in the range 3.56–7.39 GHz (mode 1), and when the PIN diode is turned OFF (mode 2), it operates in the ranges 1.69–1.93 GHz (lower band) and 3.44–6.06 GHz (upper band). The characteristics of the reconfigurable bandstop filter for both ON conditions in mode 1 and OFF conditions in mode 2 of the PIN diode are presented in Figure 8, while return loss and insertion loss values are listed in Table 1. It is observed that in both modes, the bandstop filter operates well at 4.75 GHz. In addition, mode 2 is able to support 1.77 GHz for the bandstop filtering operation. The filter equivalent circuits for mode 1 and mode 2 are shown in Figure 9 and Figure 10, respectively. They were developed by relating the filter structure with microstrip discontinuities, such as T junctions, bends, gaps, and open-end structures. The values of different elements were evaluated following the equations mentioned in [23]. An ADS platform was used to optimize LC element values as per the desired filter operations under mode 1 and mode 2. It was found that the equivalent circuit results closely follow the HFSS simulation results in terms of S-parameters for both modes, as shown in Figure 11. The lumped element values for mode 1 and mode 2 are listed in Table 2.

**Figure 12:**
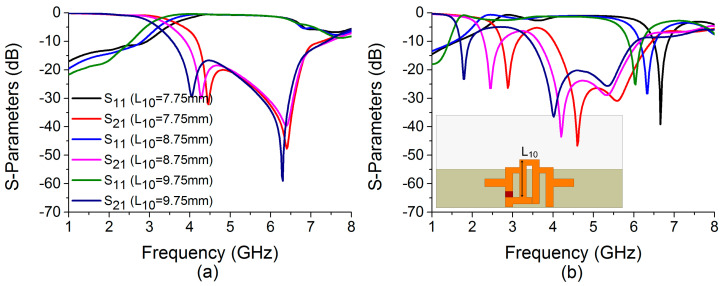
Influence of L_10_ on S-parameters in (**a**) mode 1 and (**b**) mode 2.

A small portion of the decoupling network (W_11_) is not above the ground plane. This part is located outside the ground plane because the vertical length of one filter arm L_10_ is extended to 9.75 mm. If the value of L_10_ is reduced to bring the complete decoupling structure above the ground plane, the bandstop filter in mode 2 loses its operating frequency of 1.77 GHz. This is depicted in Figure 12. Therefore, L_10_ is set to 9.75 mm for the decoupling network to function properly in mode 2.

**Figure 13:**
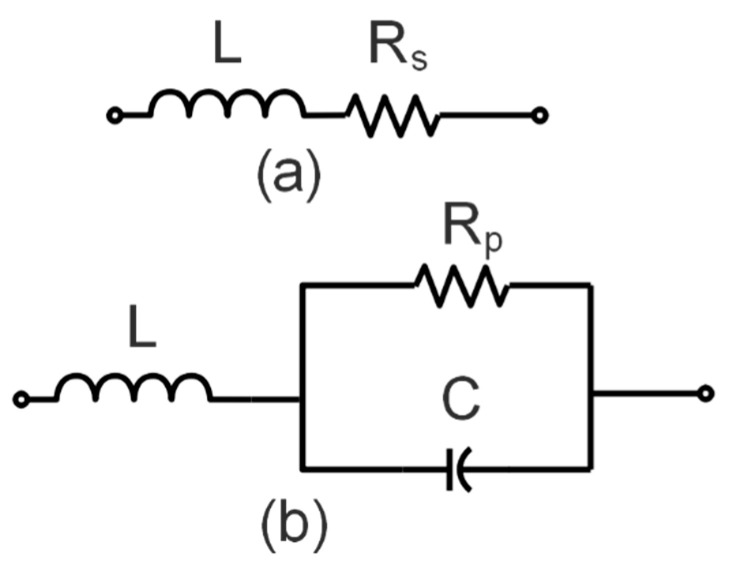
Models of equivalent circuits for the PIN diode in (**a**) ON state (**b**) OFF state.

The SMP 1320-079 LF PIN diode is used for the switching function of the reconfigurable filter. The PIN diode depicted in Figure 13 is modeled as a series RL circuit in the ON state and a combination of parallel series RLC circuits in the OFF state. A supply of 9 volts is applied to bias the PIN diode. The PIN diode has an ON state resistance and inductance of Rs = 0.75 Ω and L = 0.7 nH, respectively. In the OFF state, the PIN diode has C = 0.23 pF shunt capacitance and Rp = 0.4 MΩ reverse resistance, with 0.16 µH series inductance, as illustrated in Figure 13. Thus, two configurations of bandstop filters were made to design decoupling networks for two resonating frequencies of the two-port antenna designed in this phase.

### 2.4. MIMO Antenna with Reconfigurable Bandstop Filter Design

**Figure 14:**
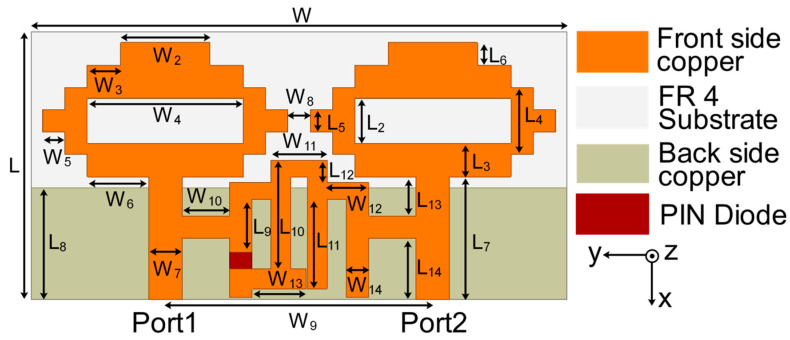
Complete two-port MIMO antenna structure with decoupling network.

**Figure 15:**
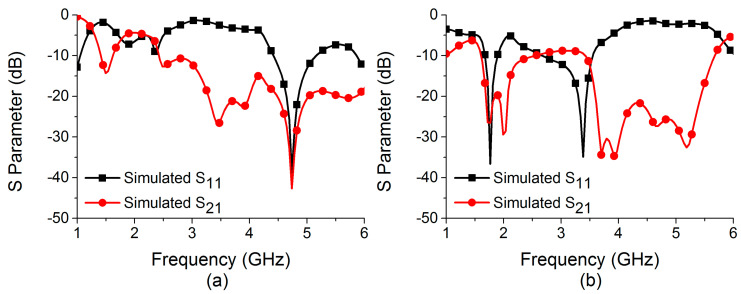
Simulated S-parameters of MIMO antenna in (**a**) ON state and (**b**) OFF state.

**Figure 16:**
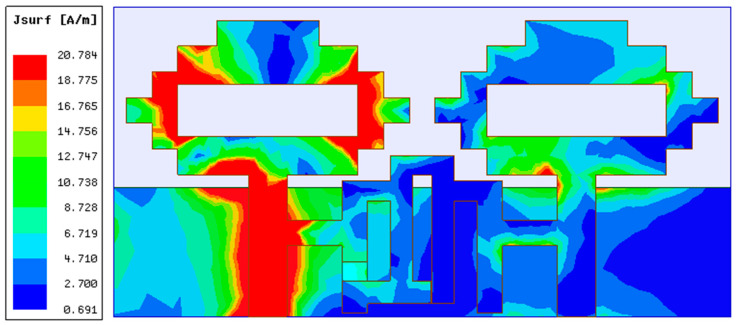
Two-element MIMO antenna surface current distribution at 4.75 GHz.

**Figure 17:**
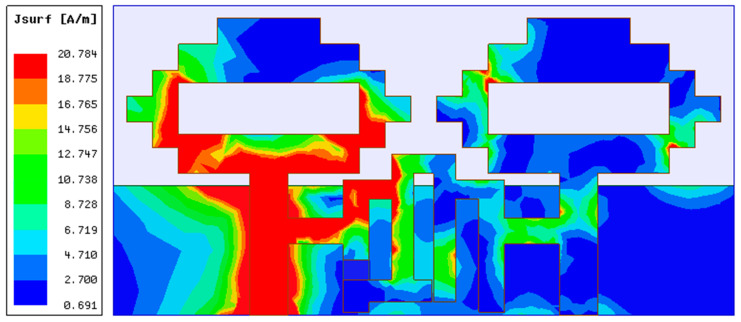
Two-element MIMO antenna surface current distribution at 1.77 GHz.

In the last stage, the developed reconfigurable filter from phase C was deployed as a decoupling network connecting two antenna elements created in phase B, as shown in Figure 14. With the incorporation of a decoupling network, the switchable bandstop filter is capable of eliminating mutual coupling at 4.75 GHz under ON conditions of the PIN diode (Mode 1) and 1.77 GHz under OFF conditions of the PIN diode (Mode 2), as indicated by the S-parameters of the MIMO antenna shown in Figure 15. A reduction in mutual coupling at 4.75 GHz and 1.77 GHz can be observed from the surface current distributions displayed in Figure 16 and Figure 17, respectively. The 4.75 GHz resonance in mode 1 is caused by actual radiation from the antenna element, whereas the 1.77 GHz resonance in mode 2 is caused by the reduced effective length of the current path in the decoupling network due to the PIN diode being turned off. The change in the current path length in the diode’s OFF condition in the bandstop filter also causes the 4.75 GHz resonance to deviate from its initial position in mode 2. Though the filter works well at 4.75 GHz in mode 2, it is unable to reduce the mutual coupling at this frequency. Therefore, it cannot be considered an operating frequency for the MIMO antenna. Thus, the switchable bandstop filter isolation technique was employed in the construction of the two-port reconfigurable MIMO antenna. The switching function of the reconfigurable filter is performed by a single PIN diode, which makes the reconfiguration circuitry simple. When the PIN diode is turned on, the designed reconfigurable structure functions as a 4.41–5.16 GHz two-port antenna system, whereas when it is turned off, it operates as a 1.67–1.9 GHz two-port antenna system. The size and design characteristics of the proposed MIMO antenna were optimized using simulations on the High-Frequency Structure Simulator (HFSS) platform. Additional design parameters for the final structure of the two-port MIMO antenna include: W = 48, L = 24, L_13_ = 3.5, and L_14_ = 5.5 (unit: mm).

## 3. Parametric Analysis

A thorough parametric study was carried out to determine the size of different structural components of the antenna structure. W_4_, L_2_, W_7_, W_9_, L_9_, and L_13_ all play important roles in enhancing impedance matching and boosting isolation for two-port MIMO antenna structures.

**Figure 18:**
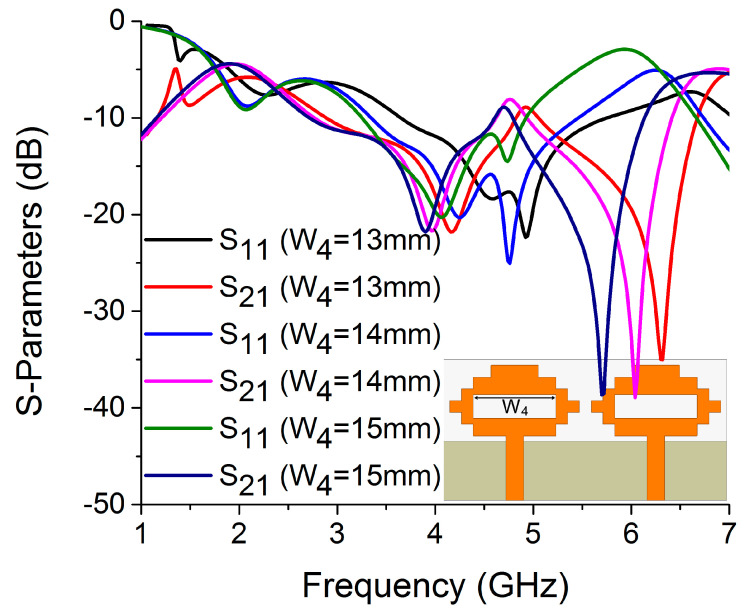
Influence of W_4_ on S-parameters.

**Figure 19:**
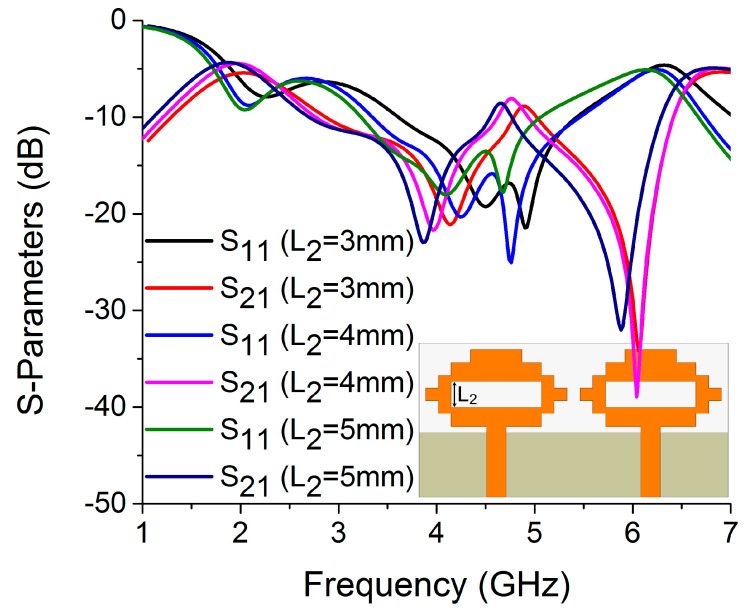
Influence of L_2_ on S-parameters.

**Figure 20:**
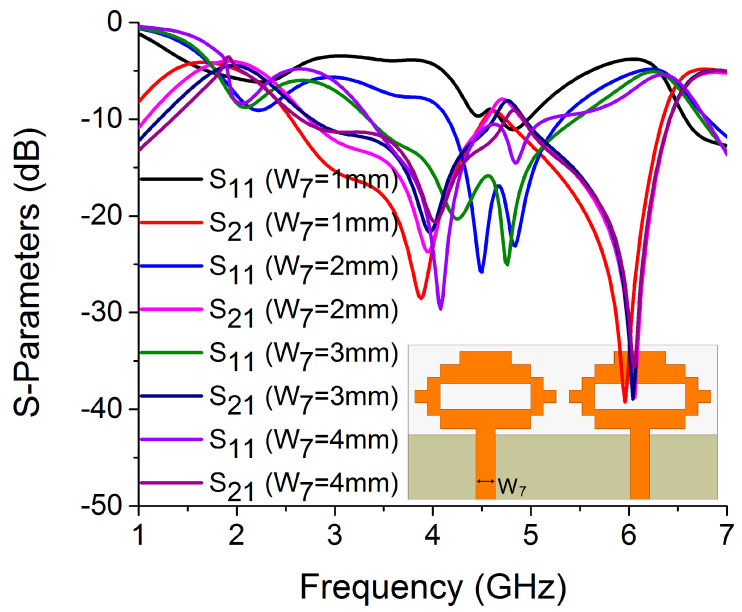
Influence of W_7_ on S-parameters.

In this antenna structure, variation in the current path length on W_4_ and L_2_ causes a significant shift in resonance frequency. The values of W_4_ and L_2_ were determined to be 14 mm and 4 mm, respectively, to optimize the resonance and isolation at 4.75 GHz, as illustrated in Figure 18 and Figure 19. The width of the antenna feed line (W_7_) also had an impact on the resonating frequency and isolation. The optimum result was obtained at 3 mm by varying the W_7_ length, as shown in Figure 20.

**Figure 21:**
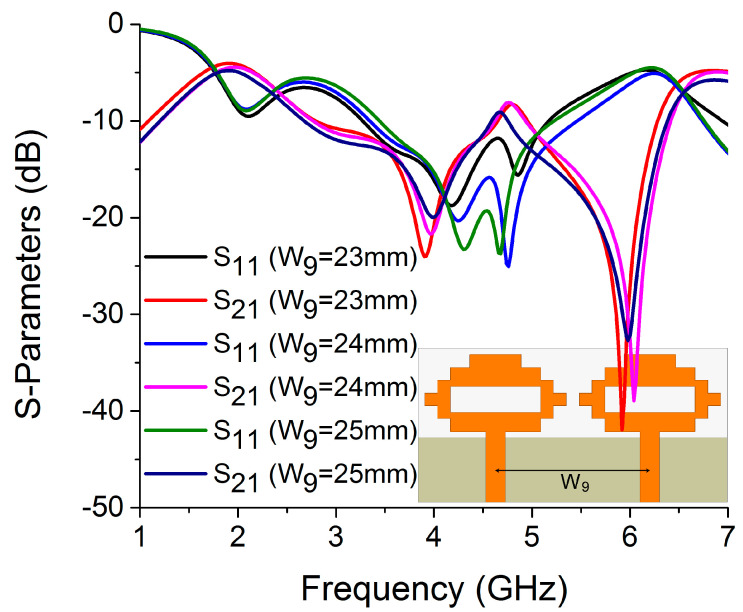
Influence of W_9_ on S-parameters.

Isolation (S_21_) is influenced by the separation between two antenna elements. In terms of edge-to-edge gap (W_8_) and center-to-center gap (W_9_), the spacing between antenna elements was optimized. The isolation between two antenna elements increases as their separation increases, which can be seen in Figure 21. However, increasing the space between antenna parts must be kept to a minimum in order to achieve a compact size. Therefore, to improve antenna isolation while retaining overall size, a decoupling network is needed.

**Figure 22:**
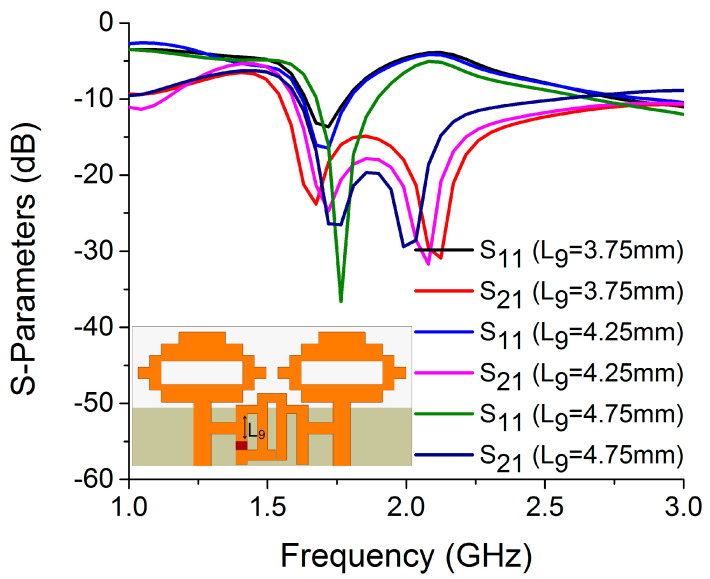
Influence of L_9_ on S-parameters.

An investigation was carried out to determine the impact of L_9_ length on resonance and isolation. Figure 22 shows how the resonance is affected by the position of the switch in the bandstop filter. It was found that only with L_9_ = 4.75 mm were both resonance and isolation achieved at the same frequency.

**Figure 23:**
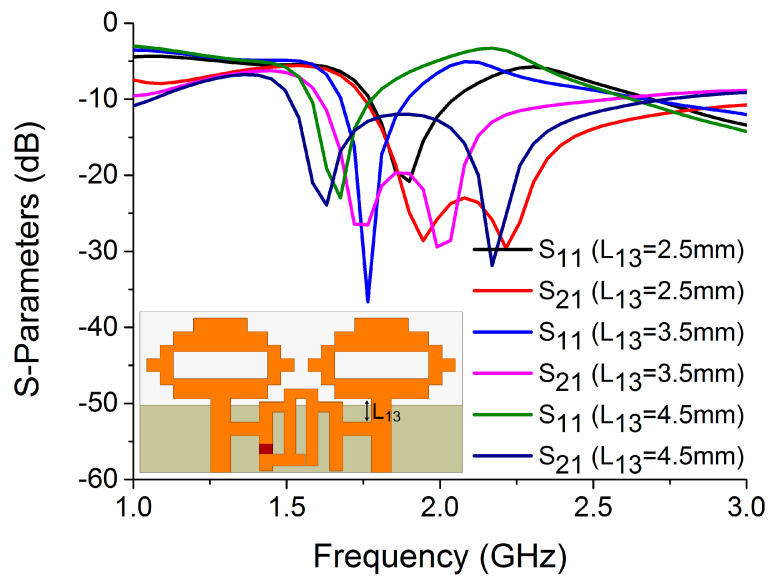
Influence of L_13_ on S-parameters.

In order to achieve deep resonance and high isolation, L_13_ was also found to be an important parameter for mode 2 operation. As illustrated in Figure 23, L_13_ = 3.5 mm produced the best results, while values other than 3.5 mm degraded performance.

## 4. Results and Discussion

**Figure 24:**
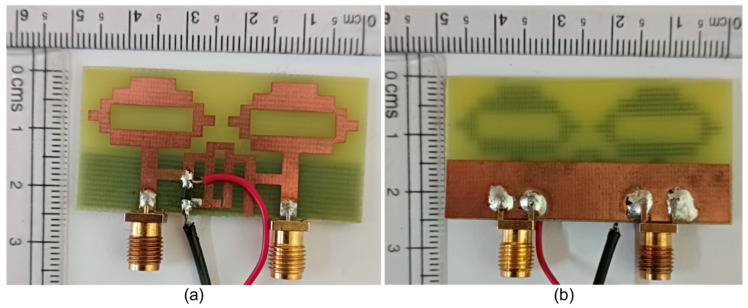
Reconfigurable two-port MIMO antenna prototype: (**a**) top view and (**b**) bottom view.

To validate the simulated results by measurements of the current approach, a prototype of the required MIMO antenna was manufactured and excited with SMA connectors, as can be seen in Figure 24, and its characteristics were examined after inserting a single PIN diode at a suitable place with proper DC biasing.

The following are three different types of experiments that were carried out using both switching options:

A. Scattering parameters;
B. Gain, efficiency, and radiation patterns;
C. Diversity parameters.

The outcomes of the three types of experiments are described below.

### 4.1. Scattering Parameters

**Figure 25:**
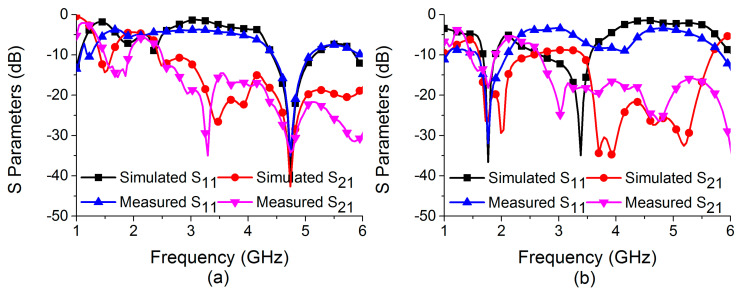
Simulated and measured S-parameters of the reconfigurable MIMO antenna in (**a**) ON state and (**b**) OFF state.

The MIMO antenna simulation results for return loss and insertion loss under ON and OFF conditions are compared to measured results, as shown in Figure 25. Thus, switching the ON/OFF conditions of the PIN diode in the bandstop filter allows the MIMO antenna to be resonant at 4.75 GHz for mode 1 operations and 1.77 GHz for mode 2 operations, respectively. The integrated PIN diode switchable bandstop filter is capable of providing strong isolation for both MIMO antenna resonating frequencies. At operating frequencies of 4.75 GHz and 1.77 GHz, the operational bandwidth is 750 MHz (4.41–5.16 GHz) and 230 MHz (1.67–1.9 GHz), respectively. There are discrepancies between simulation and measurement results. This may be due to fabrication errors, SMA connector soldering, or biasing circuitry. The correlation between simulation and measurement results is weaker in mode 2 operation than in mode 1. The mode 2 operation is primarily an outcome of mutual interaction between two antenna elements. The presence of DC bias wires may have strongly affected the mutual interaction in the fabricated model.

### 4.2. Gain, Efficiency, and Radiation Patterns

**Figure 26:**
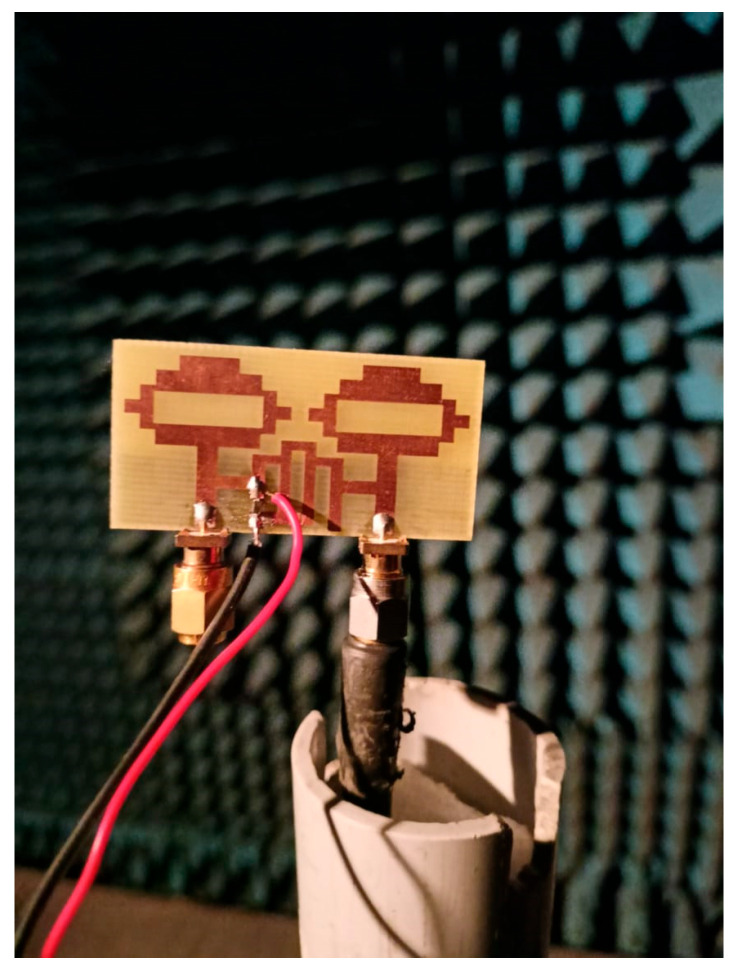
Anechoic chamber measurement setup for measuring the radiation pattern.

**Figure 27:**
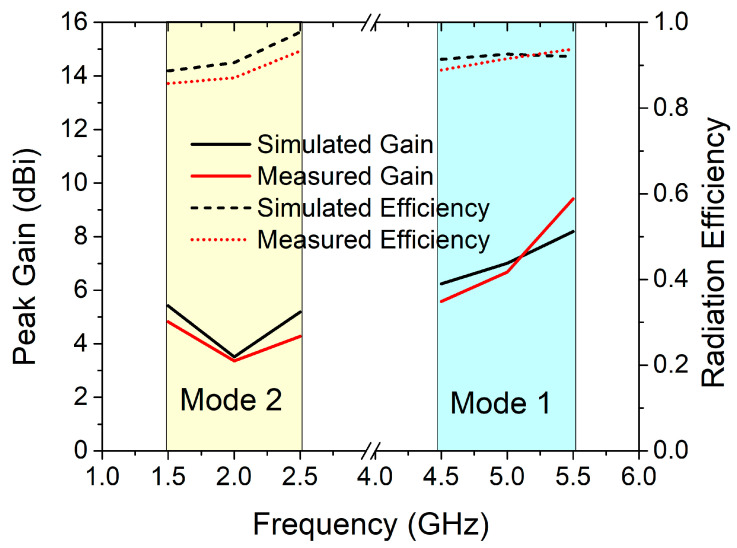
Peak gain and radiation efficiency in both modes.

In an anechoic chamber, the proposed antenna was tested for gain, efficiency, and radiation patterns. The measurement setup for the experiments is shown in Figure 26. Under the ON and OFF conditions of the reconfigurable MIMO antenna, the peak gain and antenna efficiency are shown in Figure 27. This ensures that the proposed MIMO antenna is used in the cognitive communication system to support primary and secondary communication. The recommended reconfigurable MIMO antenna seems to have a peak gain of 6.63 dBi in mode 1 and 4.41 dBi in mode 2, with an antenna efficiency of 92.04 percent and 89.64 percent, respectively. In the experimental environment, the presence of two DC lines in the prototype, as illustrated in Figure 24, slightly reduces the gain and efficiency of the proposed antenna.

**Figure 28:**
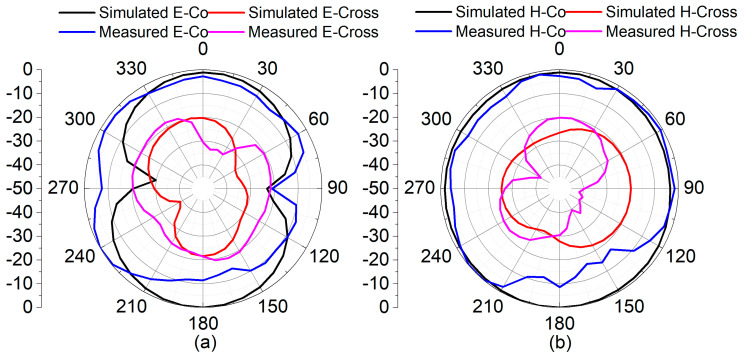
Simulated and measured normalized radiation patterns at 4.75 GHz: (**a**) E-Plane and (**b**) H-Plane.

**Figure 29:**
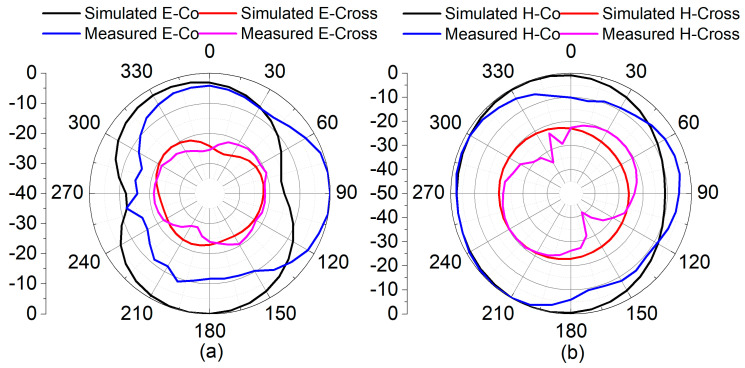
Simulated and measured normalized radiation patterns at 1.77 GHz: (**a**) E-Plane and (**b**) H-Plane.

In the simulation and measurement platform, the radiation pattern of the proposed reconfigurable MIMO antenna was observed for both modes, as shown in Figure 28 and Figure 29 with E-plane and H-plane co-polarization and cross-polarization, respectively. The differences between the maximum and minimum gain from both radiation patterns for the azimuthal plane at both frequencies are greater than 10 dB. The differences in radiation patterns observed between simulation and measured results could be attributed to the influence of biasing circuitry in the prototype model during measurements. Nonetheless, it ensures adequate radiated power for the surrounding region for both frequency bands.

### 4.3. Diversity Parameters

To guarantee that the MIMO antenna makes effective use of the surrounding environment, performance factors for diversity, including the envelope correlation coefficient (ECC), diversity gain (DG), total active reflection coefficient (TARC), mean effective gain (MEG), and channel capacity loss (CCL), should be investigated along with the scattering matrix and radiation pattern.

The envelope correlation coefficient (ECC) is a parameter that describes how isolated or correlated communication channels are. In an ideal situation, ECC should be zero, but in practice, anything less than 0.5 is acceptable. The ECC was calculated using the antenna’s far-field three-dimensional radiation pattern [24].

(1) $$\mathrm{ECC}=\frac{{\left|\iint_{4\pi }\left[\bar{F_{1}}\left(\theta ,\phi \right)\cdot \bar{F_{2}}\left(\theta ,\phi \right)d\Omega \right]\right|}^{2}}{\iint_{4\pi }{\left|\bar{F_{1}}\left(\theta ,\phi \right)\right|}^{2}d\Omega \iint_{4\pi }{\left|\bar{F_{2}}\left(\theta ,\phi \right)\right|}^{2}d\Omega }$$

**Figure 30:**
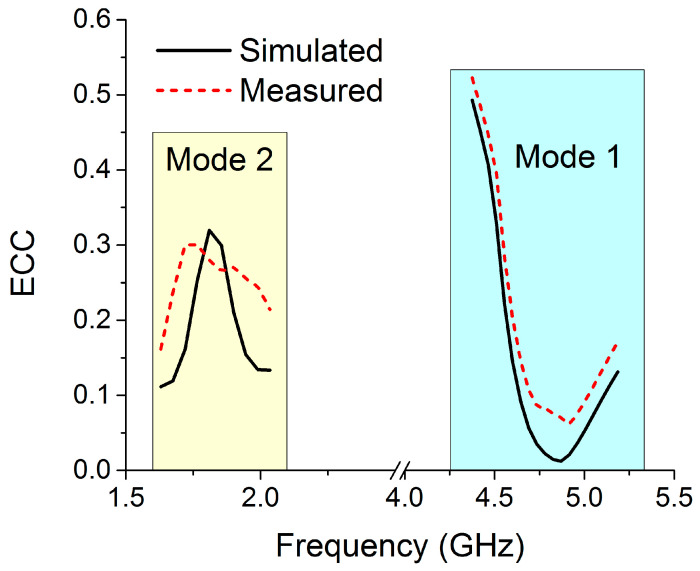
ECCs for the MIMO antenna in both modes.

At the intended frequencies, the ECCs for mode 1 and mode 2 of the proposed antenna are significantly below the permissible limit, as demonstrated in Figure 30.

The MIMO design must have a high diversity gain (DG), approaching 10 dB in the operational bandwidth, in order to ensure that MIMO systems are of high quality and reliable. Equation (2) [25] can be used to determine DG using the ECC value of the MIMO antenna.

(2) $$DG=10\times \sqrt{1-{\mathrm{ECC}}^{2}}$$

**Figure 31:**
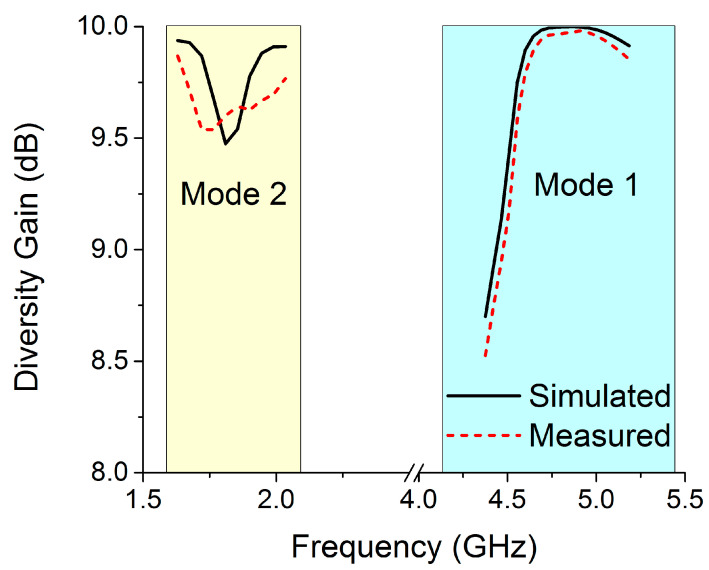
DG for the MIMO antenna in both modes.

The *DG* of the proposed switchable MIMO structure is depicted in Figure 31. The DG of the reconfigurable MIMO antenna is approximately 10 dB for both modes within the required bands, as can be seen.

The total active reflection coefficient (TARC) value can be calculated using the S-parameters of the MIMO antennas. The range of TARC values is 0 to 1. A TARC value of ‘0′ implies that the antenna radiates all available input power [26]. Therefore, at the operational frequency, the TARC value of the MIMO antenna should be close to ‘0′. For antennas with high efficiency, TARC can be calculated using a general formula based on measured S-parameters, which is defined by

(3) $$\Gamma_{a}^{t}=\frac{\sqrt{\sum_{i=1}^{N}{\left|b_{i}\right|}^{2}}}{\sqrt{\sum_{i=1}^{N}{\left|a_{i}\right|}^{2}}}$$

The TARC equation can also be calculated using the scattering matrix values given in Equation (4) [27].

(4) $$\Gamma_{a}^{t}=\sqrt{\frac{\left(\left({\left|S_{11}+S_{12}e^{j\theta }\right|}^{2}\right)+\left({\left|S_{21}+S_{22}e^{j\theta }\right|}^{2}\right)\right)}{2}}$$

**Figure 32:**
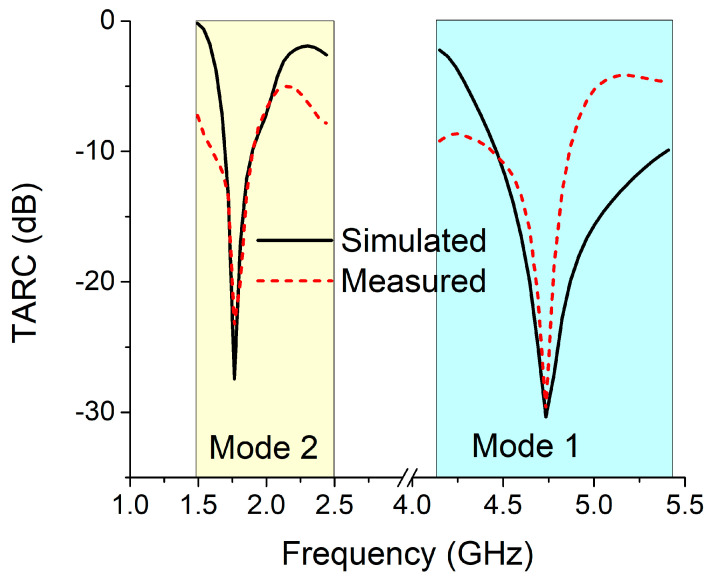
TARC for the MIMO antenna in both modes.

Figure 32 depicts the TARC values for the proposed reconfigurable MIMO antenna in both modes. It can be seen that the MIMO antenna has a sufficient TARC value for both modes at the operating frequency.

**Figure 33:**
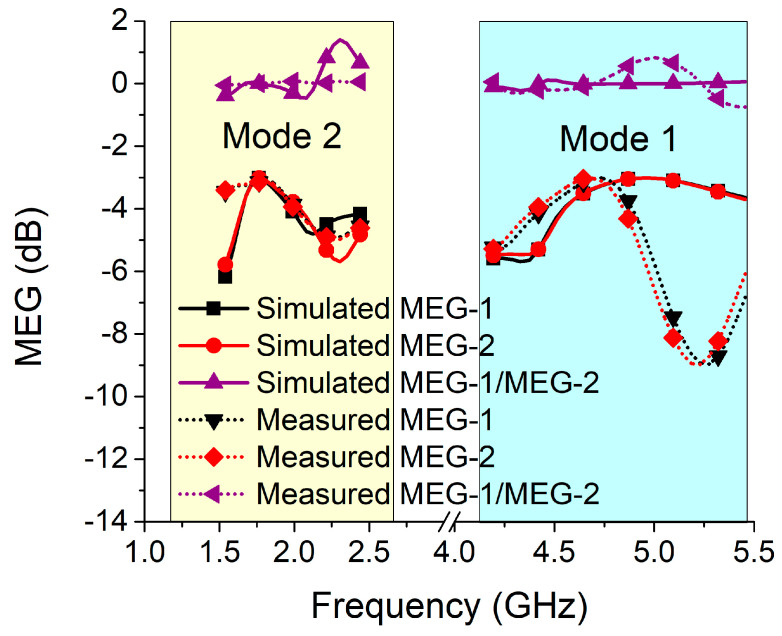
MEG for the MIMO antenna in both modes.

The gain of a MIMO antenna is calculated using mean effective gain (MEG), which takes into consideration the influences of the surrounding environment. The MEG analysis for the proposed MIMO antenna was carried out at port 1 and port 2 for both modes, as illustrated in Figure 33, using the formulations in Equations (5) and (6) [28].

(5) $${\mathrm{MEG}}_{1}=0.5\left[1-{\left|S_{11}\right|}^{2}-{\left|S_{12}\right|}^{2}\right]$$

(6) $${\mathrm{MEG}}_{2}=0.5\left[1-{\left|S_{12}\right|}^{2}-{\left|S_{22}\right|}^{2}\right]$$

To provide a functionable MIMO configuration with the same power level across all ports, the MEG-1/MEG-2 ratio has to be less than 3 dB. As demonstrated in Figure 33, the ratio for mode 1 and mode 2 of the proposed MIMO antenna is significantly less than 3 dB at the intended frequency.

The channel capacity loss (CCL) is a method that establishes the maximum amount of channel loss that may be tolerated by a communication channel for message transmission. For a transmission to be successful, the acceptable CCL must be kept below 0.4 bits/s/Hz. Equations (7) and (8) [29] can be used to calculate the CCL using the S-parameters.

(7) $$C_{\mathrm{loss}}=-\log_{2}\left|\varphi^{R}\right|$$

(8) $$\varphi^{R}=\left[\begin{array}{cc} \varphi_{11} & \varphi_{12}\\ \varphi_{21} & \varphi_{22} \end{array}\right]$$

where

$$\varphi_{11}=1-({\left|S_{11}\right|}^{2}+{\left|S_{12}\right|}^{2})$$

$$\varphi_{22}=1-({\left|S_{22}\right|}^{2}+{\left|S_{21}\right|}^{2})$$

$$\varphi_{12}=-\left(S_{11}^{*}S_{12}+S_{21}^{*}S_{22}\right)$$

$$\varphi_{21}=-\left(S_{22}^{*}S_{21}+S_{12}^{*}S_{11}\right)$$

**Figure 34:**
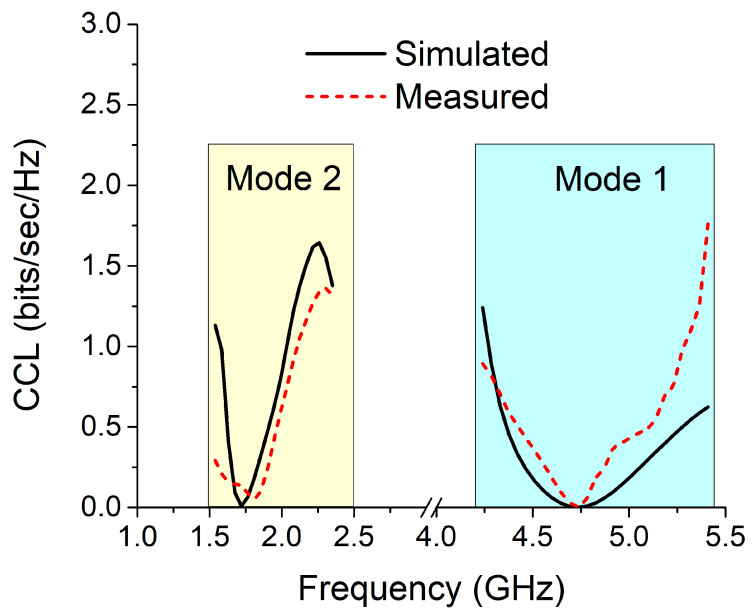
CCL for the MIMO antenna in both modes.

In both the simulation and measurement, the CCL value for both modes is significantly less than the threshold point for the intended communication frequency, as shown in Figure 34.

## 5. Comparative Analysis

Table 3 compares the proposed reconfigurable MIMO antenna to some of the two-port MIMO antennas that have previously been published. For the purposes of comparison, reconfiguration based on PIN diodes was mainly considered. The table also includes information about the isolation techniques used in the papers. It was discovered that the proposed reconfigurable MIMO antenna provides superior isolation in both mode 1 and mode 2 resonant frequencies when compared to the antennas listed in the table [16,17,19,30]. The proposed bandstop filter decoupling technique also significantly improves the isolation of reconfigurable MIMO antennas. It has a smaller size than the antennas indicated in the table, in addition to having strong isolation. Because only a single PIN diode is connected for reconfiguration, the reconfiguration circuitry of the proposed reconfigurable MIMO antenna is found to be simpler than the others listed in the table. Compared to the antennas indicated in the table, with the exception of [16], the proposed reconfigurable MIMO antenna achieves greater gain at both operational frequencies. The ECC value of the developed antenna is higher than those of the work reported in [16,17,18,30]. Despite the fact that these studies used the scattering parameter to analyze ECC, the recommended antenna uses the far-field radiation pattern to estimate ECC. The recommended MIMO antenna features a lower channel capacity loss, a more significant gain, and a simple structure as compared to previous MIMO antennas. Thus, the proposed MIMO antenna has numerous advantages over other antennas that have been published.

## 6. Conclusions

In this research work, a switchable bandstop filter was employed as a decoupling structure in the development of a compact reconfigurable MIMO antenna. The switchable bandstop filter was deployed as a decoupling network between two closely spaced monopole antennas to keep mutual coupling to a minimum at two different frequencies. At the same time, a single PIN diode was connected in the filter configuration to simplify the reconfiguration circuitry. It was possible to obtain a MIMO functioning frequency at 4.75 GHz in mode 1 and 1.77 GHz in mode 2 by controlling the ON or OFF conditions of the PIN diode, with isolation of 42.68 dB and 26.52 dB, respectively, at these frequencies. The proposed reconfigurable MIMO antenna achieved a peak gain and radiation efficiency of 6.63 dBi and 92.04 percent in mode 1 and 4.41 dBi and 89.64 percent in mode 2. Diversity parameters such as ECC, DG, TARC, MEG, and CCL were measured for both modes, as well as key antenna performance metrics. Satisfactory findings were made, including all metrics in the appropriate frequency bands. The simulation outcomes for the recommended antenna system were validated by measured results. It was observed that the proposed antennas, due to their compact size and ability to reconfigure, are suitable for cognitive radio applications.

## Figures and Tables

**Table 1:** Simulated results of filter performance.

Filter Metrics	Mode 1	Mode 2
10 dB Band (S_21_)	3.56–7.39 GHz	1.69–1.93 GHz, 3.44–6.06 GHz
Filtering frequency	4.75 GHz	1.77 GHz
Return loss (S_11_)	0.67 dB	0.86 dB
Isolation (S_21_)	18.07 dB	23.27 dB

**Table 2:** Element values of equivalent circuits.

Modes	Inductor (nH)	Capacitor (pF)
Mode 1	L1 = 0.1, L2 = 0.09, L3 = 1.86, L4 = 0.51, L5 = 0.1, L6 = 05, L7 = 0.5, L8 = 0.8, L9 = 3.7, L10 = 3.7, L11 = 31, L12 = 0.18, L13 = 0.11, L14 = 9, L15 = 0.5, L16 = 0.8, L17 = 1.2, L18 = 0.01, L19 = 0.01, L20 = 0.57, L21 = 0.15, L22 = 0.15, L23 = 0.35, L24 = 0.27, L25 = 0.92, L26 = 0.1	C1 = 0.75, C2 = 0.91, C3 = 0.55, C4 = 1.17, C5 = 1.2, C6 = 1.2, Cg = 16.68, C7 = 0.06, C8 = 0.7
Mode 2	L1 = 0.1, L2 = 0.09, L3 = 2, L4 = 0.51, L5 = 0.44, L6 = 0.4, L7 = 0.8, L8 = 0.37, L9 = 0.17, L10 = 3.3, L11 = 0.18, L12 = 0.18, L13 = 0.11, L14 = 0.57, L15 = 0.8, L16 = 1.2, L17 = 0.01, L18 = 0.01, L19 = 0.57, L20 = 0.15, L21 = 0.15, L22 = 0.86, L23 = 0.01, L24 = 0.01, L25 = 0.1	C1 = 0.55, C2 = 0.91, C3 = 0.44, C4 = 1.17, C5 = 1.25, C6 = 1.25, Cg = 16.68, C7 = 0.31, C8 = 0.7, C9 = 0.55, C10 = 0.55, Cg1 = 0.31

**Table 3:** Comparison with other decoupling network-based two-port MIMO antennas.

Ref.	Dimension	No. of Diodes	No. of Ports	Reconfiguration Technique	Isolation Technique	Frequency Reconfigurability Options	Isolation (dB)	Gain (dBi)	ECC	CCL (bits/s/ Hz)
[15]	$0.71\lambda_{0}\times 0.39\lambda_{0}$ (f = 2.35 GHz)	4	2	PIN diodes	Band-notched quarter-wave slot line on the ground plane is used between the two reconfigurable antennas.	I. 2.3–2.4 GHz II. 2.5–2.7 GHz III. 3.4–3.6 GHz	47 43 30.8	1.39 1.99 2.78	0.1108 0.0788 0.0056	10.29 10.61 11.45 (SNR Of 20 dB per port)
[16]	$0.64\lambda_{0}\times 0.32\lambda_{0}$ (f = 2.4 GHz)	2	2	PIN diodes	An isolation improvement structure (IIS) is constructed on the ground plane.	I. 2.4–2.483 GHz, 5.15–5.35 GHz II. 3.4–3.6 GHz	>20	3.81, 1.87 5.15	<0.02 <0.002 <0.002	---
[17]	$0.3\lambda_{0}\times 0.14\lambda_{0}$ (f = 3 GHz)	2	2	PIN diodes	Decoupling structure on the ground plane	I. 3–12 GHz II. 3.2–3.8 GHz	>25 dB	2.8 −1.1	<0.001	<0.5 >0.5
[18]	$0.49\lambda_{0}\times 0.16\lambda_{0}$ (f = 2.45 GHz)	4	2	PIN diodes	T slot and meander line resonator	I. 2.4–2.48 GHz, 3.4–3.6 GHz II. 5.3–5.9 GHz	>25, >28 >26	1.06, 1.02 2.46	<0.08, <0.02 <0.02	---
[19]	$0.62\lambda_{0}\times 0.44\lambda_{0}$ (f = 2.4 GHz)	2	2	PIN diodes	T slot and meander line resonator	I. 2.4, 5.2 and 5.8 GHz II. 5.2 GHz, 5.8 GH	20, 20, 20 20, 20	2.31, 3.5, 3.7 3.6, 3.75	---	---
[20]	$0.36\lambda_{0}\times 0.18\lambda_{0}$ (f = 2.5 GHz)	2	2	PIN diodes	BSF decoupling n/w	I. 3.51–3.79 GHz II. 2.43–2.6 GHz	42.77 37.49	2.92 2.98	0.067 0.154	0.04812 0.02853
[30]	$0.96\lambda_{0}\times 0.48\lambda_{0}$ (f = 2.4 GHz)	2	2	PIN diodes	Self-isolated	I. 2.0–2.7 GHz II. 3.3–4.02 GHz	16 19	3.7 4.2	0.0056 0.0009	11.1 10.2
Prop. work	$0.28\lambda_{0}\times 0.14\lambda_{0}$ (f = 1.77 GHz)	1	2	PIN diode	BSF decoupling n/w	I. 4.61–5.53 GHz II. 1.67–1.9 GHz	42.68 26.52	6.63 4.41	0.031 0.253	0.00144 0.06528

## Data Availability

Not new data was generated during the study.
